# Placental Homing Peptide-microRNA Inhibitor Conjugates for Targeted Enhancement of Intrinsic Placental Growth Signaling

**DOI:** 10.7150/thno.18845

**Published:** 2017-07-14

**Authors:** Frances Beards, Lisa E Jones, Jayne Charnock, Karen Forbes, Lynda K Harris

**Affiliations:** 1 Maternal and Fetal Health Research Centre, Faculty of Biology, Medicine and Health, University of Manchester, UK;; 2 Maternal and Fetal Health Research Centre, St. Mary's Hospital, Central Manchester University Hospitals NHS Foundation Trust, Manchester Academic Health Science Centre, Manchester M13 9WL, UK;; 3 Present address: Institute of Developmental Sciences, Faculty of Medicine, University of Southampton, Southampton, SO16 6YD, UK;; 4 Present address: Department of Biology, Faculty of Arts and Sciences, Edge Hill University, Ormskirk, L39 4QP, UK;; 5 Present address: Leeds Institute of Cardiovascular and Metabolic Medicine, Faculty of Medicine and Health, University of Leeds, Leeds, LS2 9JT, UK;; 6 Division of Pharmacy and Optometry, Faculty of Biology, Medicine and Health, University of Manchester, Manchester, M13 9PL, UK.

**Keywords:** fetal growth restriction, IGF-II, microRNA, placenta, proliferation, pregnancy.

## Abstract

Suboptimal placental growth and development are the underlying cause of many pregnancy complications. No treatments are available, primarily due to the risk of causing fetal teratogenicity. microRNAs (miRNAs) are short, non-coding RNA sequences that regulate multiple downstream genes; miR-145 and miR675 have previously been identified as negative regulators of placental growth. In this proof of principle study, we explored the feasibility of delivering miRNA inhibitors to the placentas of pregnant mice and developed novel placental homing peptide-microRNA inhibitor conjugates for targeted enhancement of intrinsic placental growth signalling. Scrambled-, miR-145- or miR-675 inhibitor sequences were synthesised from peptide nucleic acids and conjugated to the placental homing peptide CCGKRK. Intravenous administration of the miR-145- and miR-675 conjugates to pregnant C57BL/6J mice significantly increased fetal and placental weights compared to controls; the miR-675 conjugate significantly reduced placental miR-675 expression. When applied to human first trimester placental explants, the miR-145 conjugate significantly reduced placental miR-145 expression, and both conjugates induced significant enhancement of cytotrophoblast proliferation; no effect was observed in term placental explants. This study demonstrates that homing peptide-miRNA inhibitor conjugates can be exploited to promote placental growth; these novel therapeutics may represent an innovative strategy for targeted treatment of compromised placental development.

## Introduction

In the UK, more than 70,000 pregnant women per year develop serious complications such as pre-eclampsia, fetal growth restriction (FGR) and macrosomia. In the majority of cases, the underlying cause of these conditions is an abnormally developed and/or poorly functioning placenta, leading to iatrogenic preterm delivery. The consequences are twofold: in the short term, premature babies are at high risk of developing complications, and in later life affected individuals are at increased risk of developing cardiovascular or metabolic diseases 1,2. Studies in mice have demonstrated that enhancement of placental function can alleviate maternal symptoms and promote fetal growth 3,4. However, the availability of safe and effective treatments for use in human pregnancy is severely limited, because systemic administration of drugs in human pregnancy may be associated with unwanted side effects and risk of teratogenesis 5. Potential therapeutics have been identified that enhance placental growth and function, alleviate maternal symptoms and improve fetal growth in animal models of pregnancy complications 6, yet pregnant women are considered to be a high risk, low return cohort. As a consequence, there is a long standing drug drought in obstetric therapeutics: only three new drugs have been licensed for use in pregnancy in the last 20 years, two of which are used after delivery 5.

The human placenta consists of an outer syncytium, where nutrient transfer occurs, and an underlying layer of proliferating cytotrophoblasts (CTB) 7,8. The syncytial layer has a high turnover, with terminally differentiated and apoptotic elements shedding continuously into maternal circulation. To support the nutritional demands of the growing fetus, the syncytial surface area must be maintained, so CTB progenitor cells divide, differentiate and fuse with the overlying syncytium. CTB proliferation is of the utmost importance for placental growth; CTB proliferation is maximal in the first trimester of pregnancy and perturbation of proliferation is associated with the pregnancy pathologies described above 9. Abnormally high levels of proliferation can occur in women presenting with diabetes and are associated with fetal overgrowth (macrosomia) 10, whilst reduced levels are linked to fetal growth restriction and pre-eclampsia 9.

The basal rate of CTB proliferation can be augmented by exogenous growth factors and hormones including insulin-like growth factor-I (IGF-I) and IGF-II, epidermal growth factor, transforming growth factor-β, platelet-derived growth factor and the fibroblast growth factor family 11,12; however it is also regulated endogenously by the actions of specific microRNAs (miRNAs). miRNAs are short non-coding RNAs (18-22 nucleotides in length) that bind to the 3'-untranslated region (3'UTRs) of target mRNA sequences to induce direct cleavage of mRNA or indirect repression of protein translation. To date, more than 2000 mature miRNAs have been identified in humans; they are involved in gene regulation in all cells, but the function and/or targets of only a small percentage of miRNAs is currently known. Several miRNAs that are key regulators of placental growth and function have recently been identified, including some that are placental-specific, such as a large cluster identified on chromosome 19 13,14. Work by our group and others has identified numerous miRNAs, including miR-675, miR-145, let-7a, miR-377 and miR-483, that influence events in early pregnancy 15, and can either positively or negatively regulate CTB proliferation in explants of human placental tissue 16-19. Expression levels of many miRNAs are altered in pregnancy pathologies that are associated with altered placental function 20-24. Furthermore, transfection of placental explants with specific miRNA mimetics or miRNA inhibitors can enhance or inhibit CTB proliferation rates 16; thus, miRNAs represent valid therapeutic targets in human placental tissue, which can be manipulated by delivery of miRNA inhibitors or mimetics.

We have previously shown that microRNA (miR)-145 negatively regulates IGF-I-induced cytotrophoblast proliferation in human placental explants 16, and a recent publication has identified miR-675 as a regulator placental growth/function in the mouse 25. Whilst the mouse placenta is structurally somewhat different from the human placenta, enhanced trophoblast growth also translates into a larger surface area for nutrient transport 26, and interventions that increase murine placental weight are associated with an improved fetal growth trajectory 27,28. We have also shown that it is possible to use the placental homing peptide CGKRK to selectively deliver a liposomally-encapsulated payload directly to placental tissue in vitro and in vivo, minimising unwanted effects in maternal organs and fetal tissues 27. We therefore hypothesised that targeted delivery of a therapeutic that enhanced intrinsic placental growth signalling, rather than delivery of an exogenous growth signal capable of eliciting off target effects, would represent a novel treatment strategy for poor placental function. In this proof of principle study, we have explored the feasibility of using a miRNA inhibitor as putative therapeutic in pregnancy, designed placental homing peptide-microRNA inhibitor conjugates synthesised from peptide nucleic acids, and demonstrated that targeted inhibition of miR-145 and miR-675 expression within the placenta leads to enhanced CTB turnover in human first trimester explants and increased fetal and placental weights in mice.

## Materials and Methods

### Materials

Unless stated otherwise, all materials used were obtained from Sigma-Aldrich (Poole, UK).

### Homing peptide-miRNA inhibitor peptide nucleic acid conjugates

Three homing peptide-miRNA inhibitor peptide nucleic acid (PNA) conjugates were synthesised by Cambridge Research Biochemicals: (i) a scrambled miRNA inhibitor sequence conjugated to the peptide CCGKRK via a disulphide linkage (5'- ACCACGCCTCTCGCCAGTGTCAC-Cys-Cys-Gly-Lys-Arg-Lys-3'); (ii) a miR-145 inhibitor sequence conjugated to the peptide CCGKRK via a disulphide linkage (5'-CAGGTCAAAAGGGTCCTTAGGGA-Cys-Cys-Gly-Lys-Arg-Lys-3'); and (iii) a miR-675 inhibitor sequence conjugated to the peptide CCGKRK via a disulphide linkage (5'-ACCACGCCTCTCCCGGGTGTCAC-Cys-Cys-Gly-Lys-Arg-Lys-3'). A commercially available scrambled miRNA inhibitor and a selective inhibitor of miR-145, both lacking targeting peptide sequences were purchased from Exiqon and were used as negative and positive controls, respectively.

### Animal procedures

C57/BL6J mice were housed and all procedures were performed in accordance with the UK Animals (Scientific Procedures) Act 1986 at The University of Manchester. Animals had free access to food and water and were maintained on a 12:12 h light-dark cycle at 21-23°C. After mating, the presence of a copulation plug was denoted as embryonic day 0.5 (E0.5) of pregnancy.

### Assessment of fluorescent miRNA inhibitor distribution *in vivo*

A 5(6)-carboxyfluorescein (FAM)-labelled, non-targeting miRNA inhibitor (10mg/kg; Exiqon, Denmark) was administered via tail vein injection to pregnant mice. Mice received either a single injection on E12.5, with tissue collection 24h later at E13.5 (short treatment), or three separate injections on E12.5, E14.5 and E16.5, with tissue collection at E18.5 (extended treatment). Following terminal cardiac perfusion with PBS to remove unbound inhibitor, tissues were collected for analysis. Organs were snap frozen and stored at -80 ºC or fixed in neutral buffered formalin (4% (v/v); 24h). Sections of frozen tissue were fixed in ice-cold methanol (15 min), washed in PBS (2 X 5 min), mounted in Vectashield mounting medium containing DAPI (4′,6-diamidino-2-phenylindole; Vector Laboratories) and examined on a Zeiss Axiovision fluorescence microscope. Images were captured at the same exposure so that comparisons across samples could be made.

### miRNA inhibitor pilot treatment study

Mice were intravenously injected with a 5(6)-carboxyfluorescein (FAM)-labelled, non-targeting miRNA inhibitor (10mg/kg; Exiqon, Denmark), receiving either a single injection on E12.5, with tissue collection 24h later at E13.5 (short treatment), or three separate injections on E12.5, E14.5 and E16.5, with tissue collection at E18.5 (extended treatment). The following parameters were measured: fetal weight, placental weight, litter size and number of resorptions.

### Homing peptide-miRNA inhibitor conjugate treatment study

Mice were intravenously injected with 100 µl of vehicle (PBS) or 1 mg/kg of the scrambled inhibitor PNA conjugate, the miR-145 inhibitor PNA conjugate or the miR-675 inhibitor PNA conjugate on E12.5, E14.5 and E16.5 of pregnancy. Mice were sacrificed at E18.5 and the following variables measured: fetal weight, placental weight, litter size and number of resorptions. The number of animals required to observe a statistically significant change in fetal and/or placental weight following treatment (n=8 mice/group) was determined by a power calculation performed using data from previous treatment studies. Placentas were either stored in RNALater for 24h, then transferred to a -80°C freezer prior to RNA extraction, or were fixed in neutral buffered formalin (4% (v/v); 24h), subjected to routine histological processing and embedded in paraffin wax.

### Human tissue collection

Human placentas were obtained from elective medical or surgical termination of pregnancy during the first trimester (6-12 weeks) of pregnancy. Term placentas from uncomplicated pregnancies (37-42 weeks) were collected within 30 minutes of vaginal or elective caesarean delivery. Written informed consent was obtained and the study had local research ethics committee approval (13/NW/0205; 08/H1010/55(+5)).

### Villous explant culture

Villous tissue was randomly sampled and washed several times under sterile conditions, in a 1:1 ratio of serum-free Dulbecco's modified Eagle medium (DMEM) and Ham's F12 containing penicillin (100IU/ml), streptomycin (100µg/ml) and amphotericin B (2.5µg/ml) (Lonza Biosciences, UK). Explants of approximately 2mm^3^ were dissected and placed into 24-well culture plates (1/well), pre-coated with 1% (w/v) agarose. Explants were submerged in 1ml of DMEM/Ham's F12 containing glutamine (2mM), penicillin (100IU/ml), streptomycin (100ug/ml) and 10% (v/v) fetal bovine serum (Invitrogen, UK). The tissue was maintained in 95% air and 5% CO_2_ at 37ºC for up to 48h.

Placental explants were incubated with the scrambled inhibitor PNA conjugate, the miR-145 inhibitor PNA conjugate, the miR-675 inhibitor PNA conjugate, the scrambled miR inhibitor or the selective miR-145 inhibitor (50nM) for 24 or 48h. After 24h, explants were submerged in RNALater for 24h, then stored at -80ºC prior to RNA extraction. After 48h, explants were washed in PBS, fixed in neutral buffered formalin (4% (v/v); 24h), subjected to routine histological processing and embedded in paraffin wax.

### Real-time quantitative PCR

Total RNA was extracted from mouse and human placental tissue using a MirVana^TM^ miRNA Isolation Kit (Life Technologies Ltd, UK) according to the manufacturer's instructions. Total RNA was quantified using a Quant-iT Ribogreen RNA Assay Kit (Invitrogen, UK) or a Nanodrop 2000 spectrophotometer (ThermoFisher Scientific). Total RNA (10ng) was reverse transcribed using a miRCURY LNA Universal RT microRNA PCR Kit (Exiqon A/S; Denmark); quantification of miR-145 and mir-675 cDNA was performed using ExiLENT SYBR Green master mix and primers from Exiqon in a Mx3000p or a Mx3005p QPCR machine (Stratagene). miR-145-5p target sequence: 5' GUCCAGUUUUCCCAGGAAUCCCU 3' (conserved sequence between mouse and human); mmu miR-675-5p target sequence: 5' UGGUGCGGAAAGGGCCCACAGU 3'; hsa miR-675-5p target sequence: 5' UGGUGCGGAGAGGGCCCACAGUG 3'. 5-carboxyl-x-rhodamine (ROX) was used as a passive reference dye. Expression was compared to a standard curve constructed from human reference total RNA (Agilent). QPCR reactions were performed in triplicate; miRNA expression was normalised to expression of the housekeeping gene 5S ribosomal RNA using primers from Exiqon.

### Immunohistochemistry

Sections (5µm) of wax-embedded placenta were dewaxed and rehydrated using Histoclear and decreasing concentrations of ethanol, after which they were submerged in distilled water for 5 min, then microwaved at full power for 10 min in 0.01M sodium citrate buffer, pH 6.0). After cooling, the sections were washed in distilled water and endogenous peroxidase activity was quenched by incubation with 3% (v/v) hydrogen peroxide at room temperature for 10 min. Sections were washed in Tris buffered saline (TBS, 2 X 5 min) then incubated with 5% (w/v) bovine serum albumin (BSA) in TBS for 30 min at room temperature. Primary antibodies were diluted in TBS as follows: mouse anti-human Ki67 (35µg/ml, Dako); rabbit anti-mouse Ki67 (0.84µg/ml, Dako) and applied to individual sections. Isotype control mouse IgG was diluted to the same working concentration. Slides were incubated overnight at 4°C in a humidity chamber. Slides were washed in TBS (3 X 5 min), biotinylated anti-mouse secondary antibodies (Dako UK) were diluted in TBS to 4.4µg/ml, then were applied to the sections and incubated for 30 minutes at room temperature. Slides were washed again with TBS as described above, followed by incubation with avidin peroxidase (5µg/ml) for 30 minutes at room temperature. Another series of washes as above followed and sections were incubated with the chromogen diaminobenzidine (DAB 0.05% (w/v); H_2_O_2_ 0.015% (v/v)). Colour development was monitored under a light microscope and sections were then washed with dH_2_O, counterstained with Harris' hematoxylin and washed briefly in acid/alcohol solution. Finally, sections were washed in warm tap H_2_O, dehydrated in increasing concentrations of ethanol and Histoclear and mounted with DPX.

Tissue sections were stained in large batches to minimize inter-experimental variation. Slides were imaged using an Olympus Bx41 light microscope and Image ProPlus 6.0 imaging software; exposure times were matched at image capture. The number of proliferating cytotrophoblasts in human placental explants was counted manually using blinded samples. The proportion of Ki67-positive cells and the area of the junctional zone and labyrinth within each mouse placenta were quantified using HistoQuest image analysis software.

### Data Analysis

Data were analysed using GraphPad Prism software (Version 6; GraphPad, CA). Non-parametric data were expressed as medians and analysed by Kruskal-Wallis test (unpaired data) or Friedman test (paired data). Levene's test was used to assess equality of variances. Data from a minimum of 3 independent experiments is presented. Significance was taken as P<0.05.

## Results

**Figure 1:**
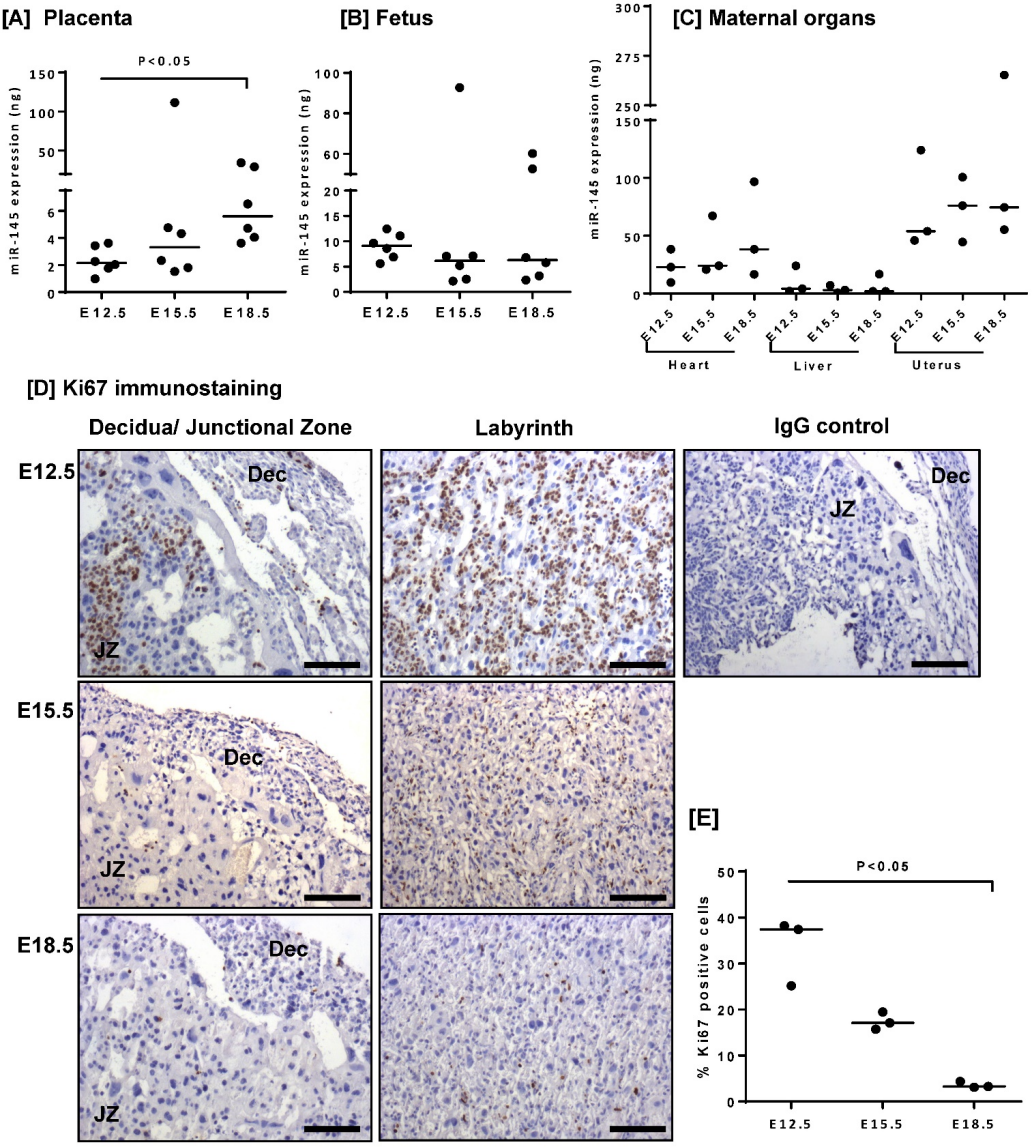
** miR-145 expression negatively correlates with proliferation rate in the mouse placenta**. miR-145 expression was assessed in **[A]** mouse placentas, **[B]** fetuses and **[C]** maternal heart, liver and uterus at E12.5, E15.5 and E18.5 by quantitative RT-PCR and normalised to expression of 5S ribosomal RNA. Median, n=3 - 6; *P<0.05; Kruskal-Wallis test with Dunn's post hoc test. **[D]** Immunohistochemical analysis of proliferating cells in mouse placental tissue stained with an antibody to Ki67 or an isotype control IgG. Diaminobenzidine (DAB) labelling, brown; nuclei counterstained with haematoxylin, blue. Scale bar represents 100µm. Images are representative of placentas from n=3 mice. JZ = junctional zone; Dec = decidua. **[E]** The percent area of Ki67-positive staining was quantified using Histoquest software. Median, n=3; *P<0.05, Kruskal-Wallis test with Dunn's post hoc test.

microRNA-145 (miR-145) expression has previously been documented in the human placenta 16; however, no corresponding data exists in the mouse. Quantitative RT-PCR analysis of mouse placental lysates confirmed miR-145 expression (Figure 1A), and demonstrated that miR-145 levels significantly increased between E12.5 and E18.5 of gestation. miR-145 was also detected in the fetus (Figure 1B), and in the maternal heart, liver and uterus (Figure 1C); however, the level of expression did not change with gestation. As observed in human placental explants 16, increased miR-145 expression correlated with a decrease in the number of Ki67-positive cells throughout the placenta and decidua (Figure 1D, E).

**Figure 2:**
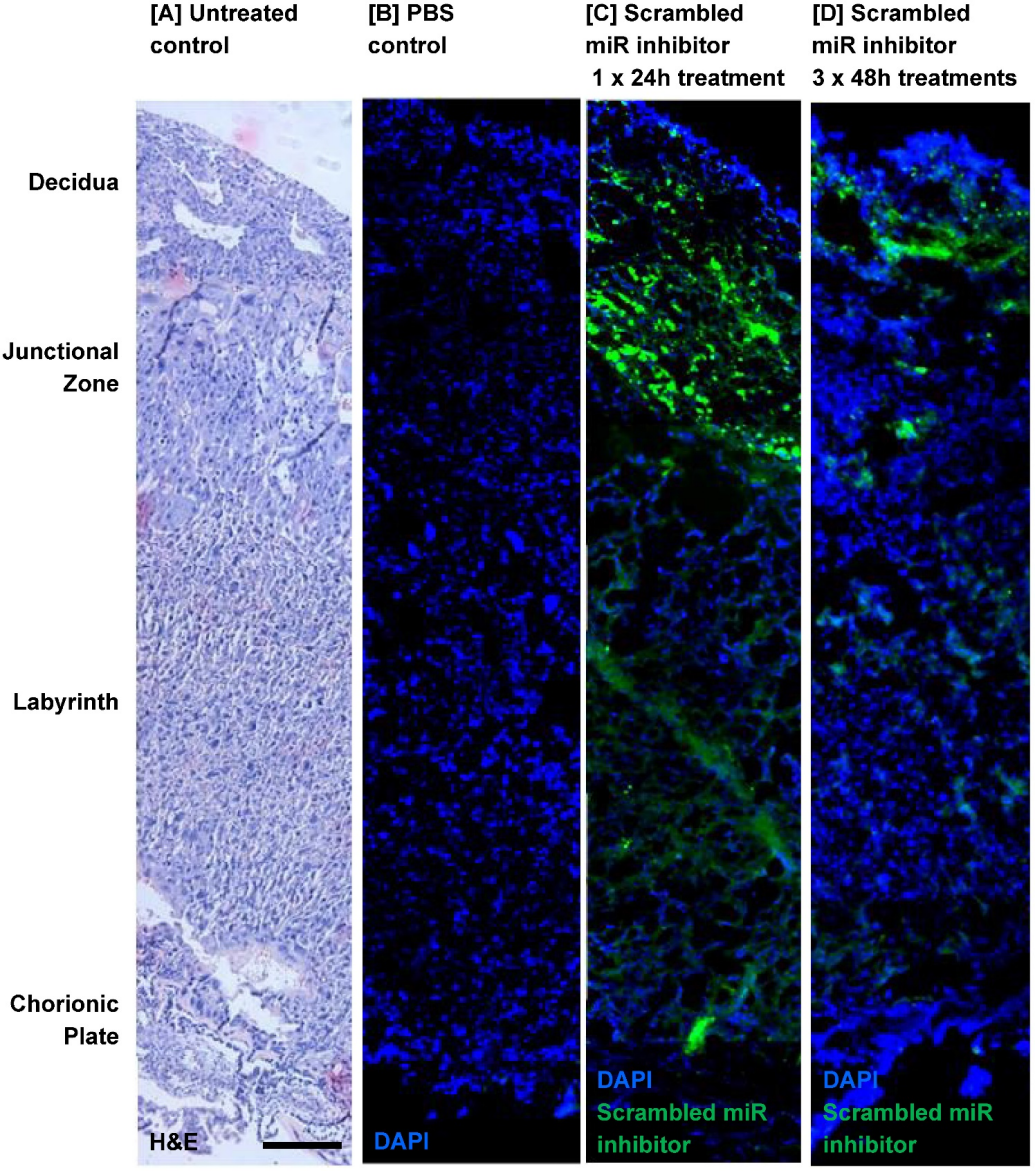
** An intravenously injected scrambled miRNA inhibitor localises to the mouse placenta**. A fluorescently labelled, scrambled miRNA inhibitor (10 mg/kg) was intravenously administered to pregnant C57/BL6J mice via the tail vein. Mice either received a single injection on E12.5, with tissue collection 24h later at E13.5 (short treatment), or three separate injections on E12.5, E14.5 and E16.5, with tissue collection at E18.5 (extended treatment). Mice were subjected to cardiac perfusion to remove unbound inhibitor, placentas were collected, fixed and frozen; miRNA inhibitor localisation was assessed by fluorescence microscopy. n=3 placentas were examined from each of N=3-5 pregnant mice. Representative images are shown. Scrambled miRNA inhibitor, green; DAPI (nuclei) blue. **[A]** A section of mouse placenta stained with haematoxylin and eosin to show tissue architecture; E13.5. **[B]** A mouse placenta collected 24h after a tail vein injection of PBS (100µl); E13.5. **[C]** A mouse placenta collected following a short-term treatment with scrambled miRNA inhibitor; E13.5. **[D]** A mouse placenta collected following a long-term treatment with scrambled miRNA inhibitor; E18.5. Scale bar represents 100µm.

**Figure 3:**
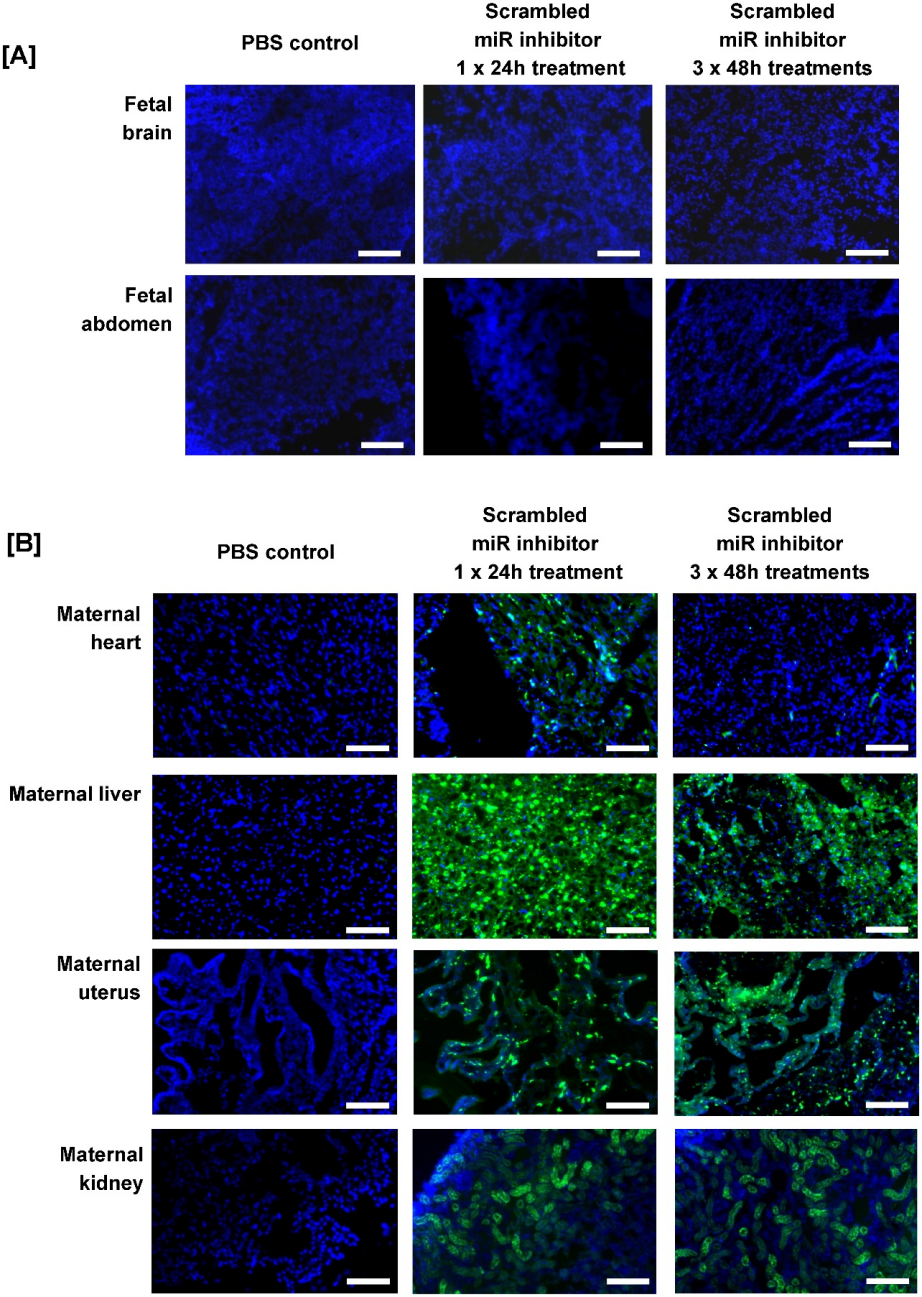
** An intravenously injected scrambled miRNA inhibitor accumulates in maternal organs. **A fluorescently labelled, scrambled miRNA inhibitor (10 mg/kg) was intravenously administered to pregnant C57/BL6J mice via the tail vein. Mice either received a single injection on E12.5, with tissue collection 24h later at E13.5 (short treatment), or three separate injections on E12.5, E14.5 and E16.5, with tissue collection at E18.5 (extended treatment). Mice were subjected to cardiac perfusion to remove unbound inhibitor, fetuses and maternal organs were collected, fixed and frozen; miRNA inhibitor localisation was assessed by fluorescence microscopy. Tissues were examined from N=3-5 pregnant mice. Representative images are shown. Scrambled miRNA inhibitor, green; DAPI (nuclei) blue. **[A]** Mouse fetuses, **[B]** Maternal heart, liver and uterus. Scale bar represents 100µm.

Before miRNA inhibitors can be considered as a clinical intervention for poor placental development, it is important to determine whether they can be successfully administered to the placenta and whether they have any detrimental effects on maternal health and/or pregnancy outcome. Pregnant C57/BL6J mice were exposed to a short-term treatment (single injection; 24h) or extended dosing (3 injections at 48h intervals) with a commercially available FAM-labelled, non-targeting miRNA inhibitor. The inhibitor was administered via intravenous injection and cardiac perfusion was undertaken prior to tissue harvest, to remove any inhibitor still freely circulating in the blood. Fluorescence microscopy revealed that the miRNA inhibitor was present in mouse placental tissue after a single 24h injection; it localised primarily to the junctional zone, but was also evident at lower and more variable levels in the labyrinth and decidua (Figure 2C). A similar pattern of localisation of reduced intensity was observed after extended treatment (Figure 2D), even though tissue was harvested two days after the final injection. No fluorescence was observed in the placentas of mice injected with a vehicle control (Figure 2B). The miRNA inhibitor was not observed in any fetal organs regardless of the treatment regimen; representative images of the fetal brain and abdomen are shown in Figure 3A. In contrast, the miRNA inhibitor was detected in a number of maternal tissues including the heart, liver, kidney and uterus (Figure 3B), indicating the possibility of significant off-target effects following systemic administration of a functional miRNA inhibitor.

Both short term and extended treatment with the scrambled miRNA inhibitor had no significant effect on median fetal or placental weights (Figure S1 A-D) and no gross fetal abnormalities were noted. In addition, the scrambled inhibitor did not alter fetal-placental weight ratio (a measure of placental efficiency; Figure S1 E, F), litter size (Figure S1 G, H) or the number of resorptions (Figure S1 I, J). These data suggest that the inhibitor was well tolerated in pregnancy.

**Figure 4:**
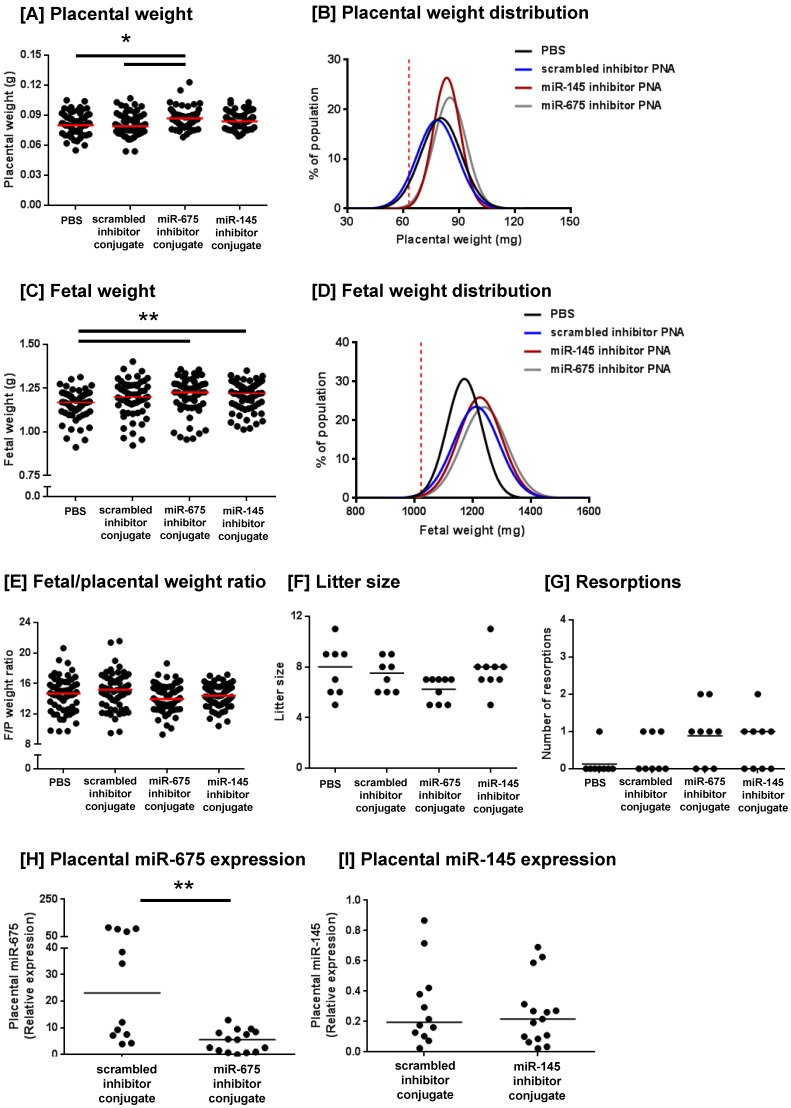
** Homing peptide-miRNA inhibitor conjugates increase fetal and placental weight in mice. **Pregnant C57/BL6J mice were intravenously injected with PBS (100µl; N=8, n=62), a targeted scrambled miRNA inhibitor conjugate (1mg/kg; N=8, n=59), a targeted miR-145 inhibitor conjugate (N=9, n=69), or a targeted miR-675 inhibitor conjugate (N=9, n=56) on E12.5, E14.5 and E16.5. Mice were sacrificed at E18.5 and the following variables measured: **[A]** placental weight; **[B]** placental weight population distribution curve; **[C] **fetal weight; **[D] **fetal weight population distribution curve; **[E]** fetal/placental weight ratio; **[F]** number of fetuses per litter, **[G]** number of resorptions per litter. *P<0.05 , **P<0.01; Kruskal-Wallis test with Dunn's post hoc test. Horizontal line represents median. Vertical dashed line in B and D represents the fifth weight centile (mg) of PBS-treated mice. **[G, H]** Placental expression of **[H]** miR-675 and **[I]** miR-145 expression was assessed by quantitative RT-PCR and normalised to expression of 5S ribosomal RNA. Median, n = 2 - 3 placentas from N = 6 mice; **P<0.01, Friedman test with Dunn's post hoc test. Data points in **A, C, E, H** and **I** represent individual conceptuses; data in **F** and **G** represent litter averages.

We have previously demonstrated that the peptide sequence CCGKRK selectively accumulates in the placentas of pregnant mice when administered intravenously and is rapidly internalised into the outer STB later of human placental explants 27. To investigate whether targeted delivery of a microRNA inhibitor could enhance placental growth in vivo, we designed CCGKRK-miRNA inhibitor conjugates which were synthesised using peptide nucleic acids. C57/BL6J mice were intravenously injected with PBS, a scrambled miRNA inhibitor conjugate (1mg/kg), a miR-145 inhibitor conjugate or a miR-675 inhibitor conjugate at three-time points during pregnancy. Mice injected with the miR-675 inhibitor conjugate exhibited a significant increase in median placental weight at E18.5, compared to mice injected with PBS or the scrambled inhibitor conjugate (Figure 4A). The miR-145 inhibitor conjugate did not significantly alter median placental weight, but it appeared to normalise placental weight, such that there were fewer of the heaviest and lightest placentas within that treatment group (Figure 4E). Levene's test for homogeneity of variance confirmed that the miR-145 inhibitor conjugate significantly reduced the variance in placental weights, compared to mice treated with PBS (P<0.05). Analysis of placental weight distribution indicated that 6 placentas weighed below the 10^th^ centile in PBS treated mice, and 5 placentas weighed below the 10^th^ centile in mice treated with the scrambled inhibitor conjugate, but no placentas fell below the 10^th^ weight centile in either miR-145 or miR-675 inhibitor conjugate-treated mice, suggestive of a growth-promoting effect. Placental weight distributions are shown in Figure 4B.

The miR-145 and miR-675 inhibitor conjugates also significantly increased median fetal weight at E18.5, compared to mice injected with PBS (Figure 4C); however, fetal weight distribution curves showed that the number of fetuses falling below the 10^th^ centile remained unchanged. Whilst the scrambled inhibitor conjugate did not significantly alter median fetal weight, it did alter fetal weight distribution; the reason for this is currently unknown. No inhibitor significantly altered the median fetal/placental weight ratio, a measure of placental efficiency; however, the miR-145 inhibitor conjugates appeared to normalise efficiency, such that fewer placentas exhibited extremes of efficiency (Figure 4E). Levene's test for homogeneity of variance confirmed that treatment with miR-145 inhibitor conjugate significantly reduced the variance in fetal/placental weight ratio, compared to that of mice treated with PBS (P<0.05) or the scrambled inhibitor conjugate (P<0.05). No PNA inhibitor conjugate significantly altered litter size (Figure 4F), nor the number of resorptions per litter (Figure 4G), demonstrating that this novel treatment was well tolerated in mice.

**Figure 5:**
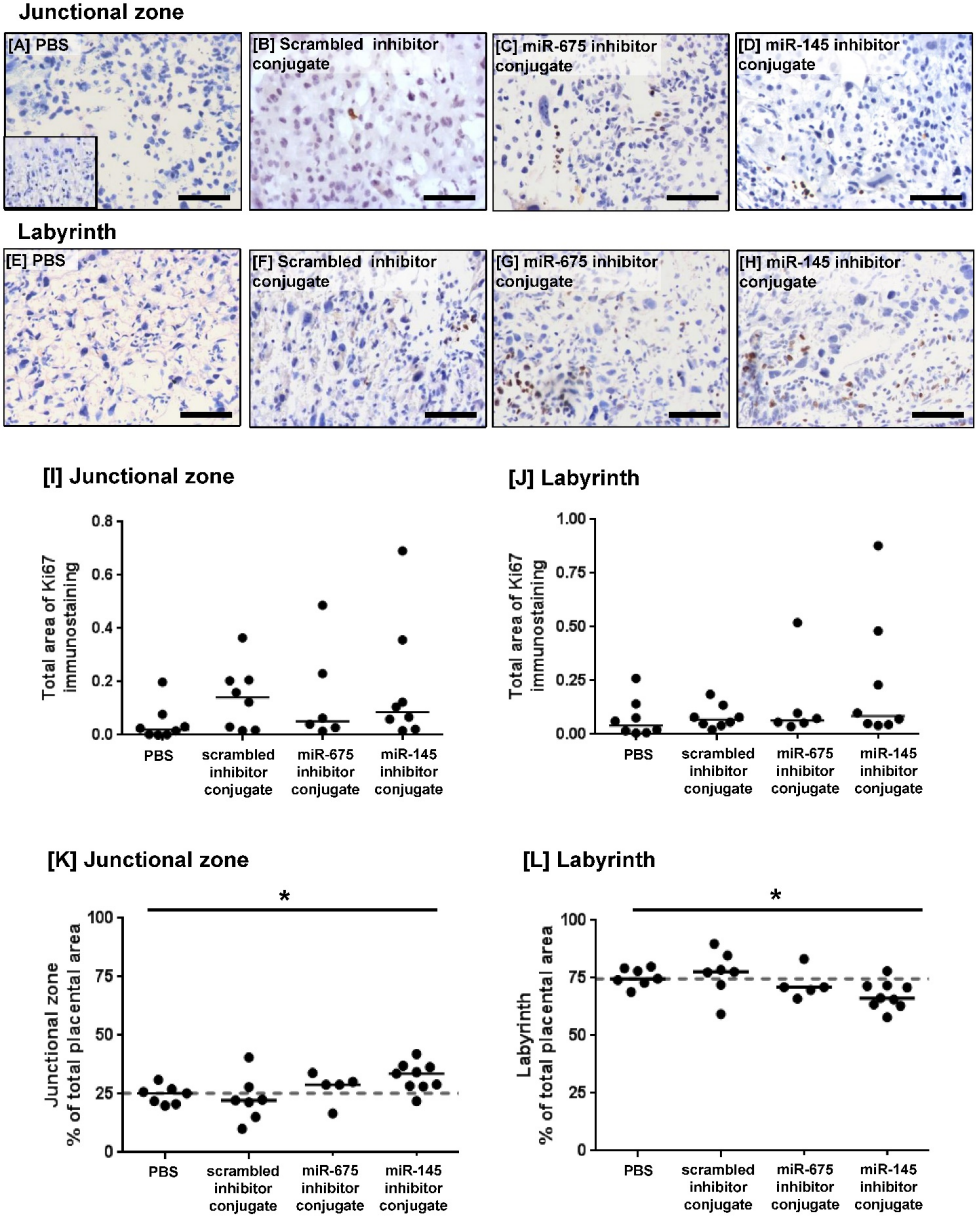
** Homing peptide-miRNA inhibitor conjugates do not increase the rate of proliferation in the mouse placenta at E18.5.** [A-H] Pregnant C57/BL6J mice were intravenously injected with PBS (100µl), a targeted scrambled miRNA inhibitor conjugate (1mg/kg), a targeted miR-145 inhibitor conjugate or a targeted miR-675 inhibitor conjugate on E12.5, E14.5 and E16.5. Mice were sacrificed at E18.5. The extent of proliferation was assessed by immunostaining mouse placentas with an antibody to Ki67 or an isotype control IgG (inset). Diaminobenzidine (DAB) labelling is shown in brown, nuclei were counterstained with haematoxylin (blue). Scale bar represents 100µm. [I, J] The total area of Ki67 immunostaining was quantified using Histoquest software. Median, n=8. [K, L] The total area of the junctional zone and labyrinth were quantified using Histoquest software, and expressed as percentage of the total placental area (median, n=5-8). *P<0.05, Kruskall Wallis test.

PCR analysis of miRNA expression in placentas harvested at E18.5 showed that treatment with the miR-675 inhibitor conjugate significantly reduced miR-675 expression (Figure 4G), but median placental miR-145 expression was not significantly changed at this time point (Figure 4H). The extent of cell proliferation in the harvested mouse placentas was assessed by Ki67 immunostaining and quantified using HistoQuest image analysis software. The total area of positive staining was variable throughout both the junctional zone and the labyrinth (Figure 5A-H) and there was no significant difference in proliferation between treatment groups at E18.5 (Figure 5I, J). Quantification of the relative areas of the junctional zone and labyrinth using HistoQuest highlighted a significant difference in median area of both zones across all treatment groups; however, post-hoc testing did not did not reveal a significant difference in median area between individual treatments (Figure 5K, L).

**Figure 6:**
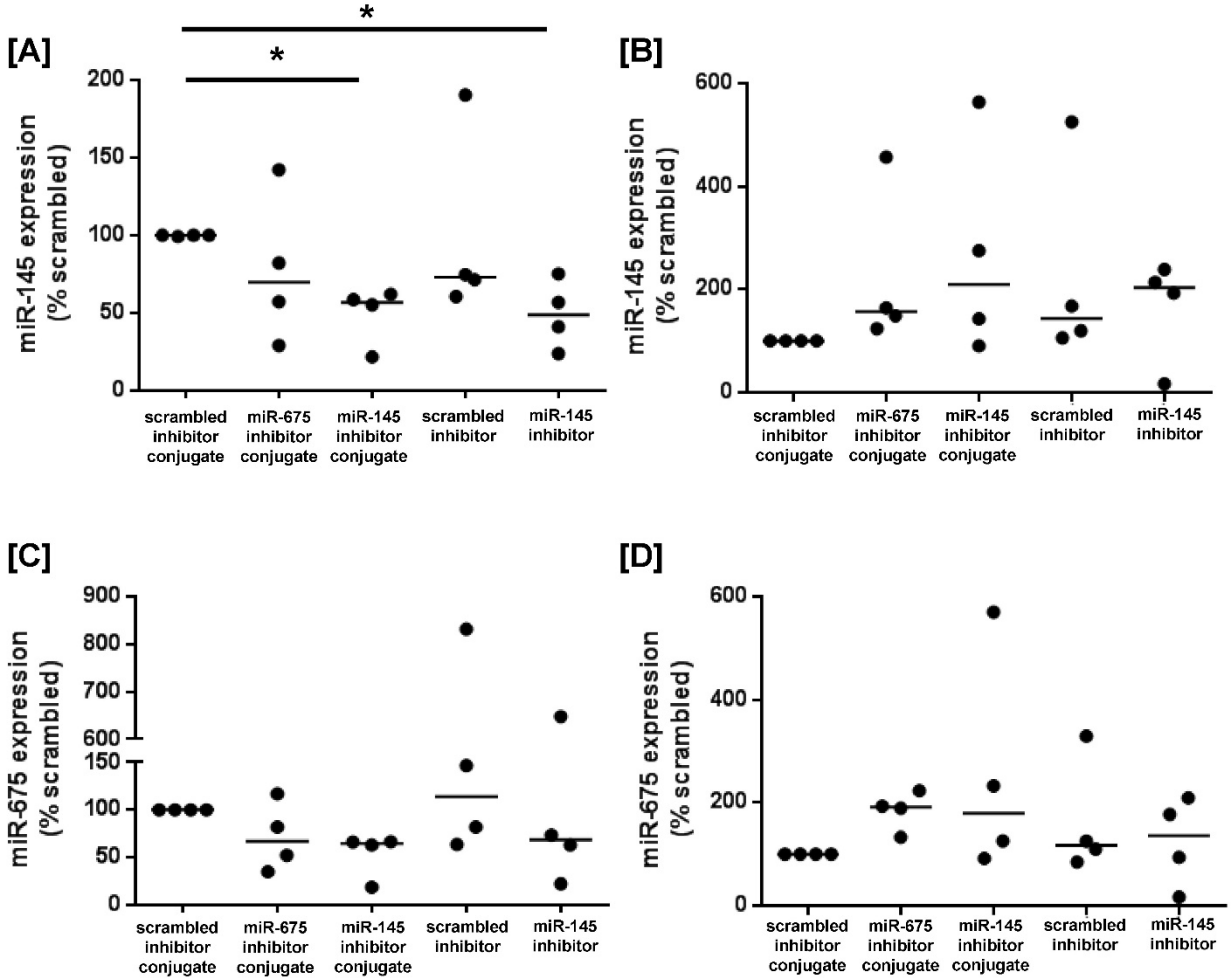
** Homing peptide-miRNA inhibitor conjugates reduce miR-145 expression in first trimester placental explants**. **[A, C]** First trimester and **[B, D]** term placental explants were incubated with either a targeted scrambled inhibitor conjugate, a targeted miR-145 inhibitor conjugate, a targeted miR-675 inhibitor conjugate, a scrambled miRNA inhibitor lacking a targeting sequence or a miR-145 inhibitor lacking a targeting sequence (50nM; 24h). **[A, B]** miR-145 or **[C, D] **miR-675 expression was assessed by quantitative RT-PCR (median, n=4). *P<0.05, Friedman test.

To determine whether miRNA inhibitor conjugates could be used to promote growth signalling in human placental tissue, first trimester and term placental explants were cultured with scrambled-, miR-145 inhibitor- or miR-675 inhibitor conjugates, or with commercially available scrambled- or miR-145 inhibitors that lacked the CCGKRK targeting sequence. After 24h of culture, miR-145 expression was significantly reduced in first trimester explants treated with the miR-145 inhibitor conjugate, and the reduction in expression was comparable to explants treated with the non-targeted miR-145 inhibitor (Figure 6A), as previously reported 16. In contrast, there was no significant reduction in miR-145 expression in term placental explants exposed to either the targeted or non-targeted miRNA inhibitor conjugate (Figure 6B). miR-675 expression was not significantly reduced in first trimester explants incubated with the miR-675 inhibitor conjugate (Figure 6C), but this may reflect the small sample size and the inherent biological variability of human tissue samples. No effect was observed in term placental explants (Figure 6D).

**Figure 7:**
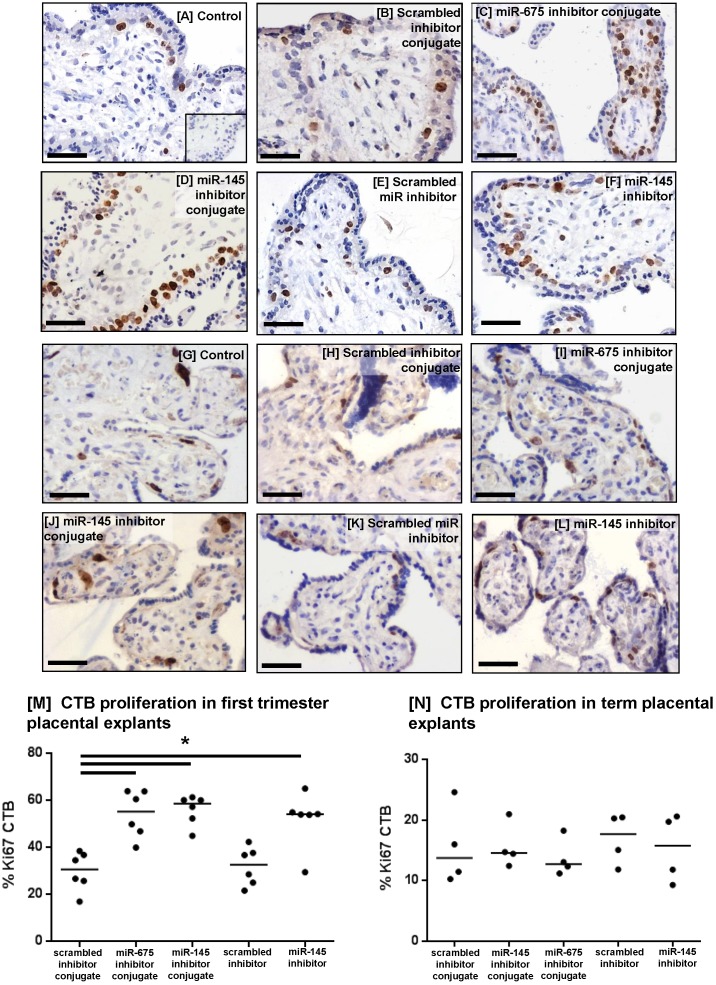
** Homing peptide-miRNA inhibitor conjugates increase cytotrophoblast proliferation in first trimester placental explants.** [A-F] First trimester and [G-L] term placental explants were incubated with either a targeted scrambled inhibitor conjugate, a targeted miR-145 inhibitor conjugate, a targeted miR-675 inhibitor conjugate, a scrambled miRNA inhibitor lacking a targeting sequence or a miR-145 inhibitor lacking a targeting sequence (50nM; 48h). Proliferation was assessed by immunohistochemical analysis of Ki67-positive cytotrophoblasts (CTB). Diaminobenzidine (DAB) labelling, brown; nuclei counterstained with hematoxylin (blue). Scale bar 50µm. Inset in [A] is control IgG. Percent of Ki67-positive CTB was quantified in [M] first trimester explants (median, n=6); *P<0.05 Friedman test, and [N] term placental explants (median, n=4).

To investigate whether the miRNA inhibitor conjugates promoted human placental cell proliferation, first trimester (Figure 7A-F) and term placental explants (Figure 7G-L) were incubated with the conjugates for 48h and the percent of Ki67-positive cytotrophoblasts (CTB) was quantified. Both miRNA inhibitor conjugates significantly enhanced CTB proliferation in first trimester explants, compared to those treated with the scrambled inhibitor conjugate (Figure 7M), and this increase was comparable to that observed with the non-targeted miR-145 inhibitor. The inhibitor conjugates had no effect on cytotrophoblast turnover in term placental explants (Figure 7N).

## Discussion

Here we describe the first report of targeted delivery of miRNA inhibitor to placental tissue, resulting in a clinically relevant enhancement of placental weight in healthy mice and enhanced CTB proliferation in human first trimester placental explants. We demonstrate expression of miR-145 in mouse placenta for the first time, propose that this molecule controls placental weight gain and validate a previous report that miR-675 is a negative regulator of murine placental growth 25. The data also show that the PNA conjugates were successfully internalised into their target cells and that the presence of the targeting sequence did not interfere with miRNA inhibitor function. Indeed, the miRNA inhibitor PNA and targeting peptide were conjugated using a disulphide linkage, so that the targeting element would be hydrolysed and released upon internalisation, and not impair binding of the inhibitor to endogenous cellular miRNAs. The CCGKRK peptide was chosen due to its effective cell penetrating properties, as well as for its ability to selectively target placental tissue 27. The conjugates retained their function in vivo, likely due to the inherent resistance of these synthetic sequences to digestion by proteases and nucleases 29. They were also well tolerated; no pathological effects were noted in dams or fetuses in this study and the lack of effect on gross placental morphology, litter size or number of pregnancy losses suggests that no overt immune response was stimulated by the conjugates. A more extensive investigation will be needed to screen for any subtle effects that may not have been detected in our pilot study; however, our current data highlights the favourable therapeutic profile of peptide-PNA conjugates in pregnancy.

A number of nanoparticle formulations have proven to be successful platforms for delivery of miRNA mimetics and antagomirs: silica nanoparticles conjugated to a disialoganglioside GD(2) antibody were used for delivery of the pro-apoptotic microRNA miR-34a to mice bearing neuroblastoma tumours 30 and peptide-decorated lipoparticles containing anti-miR-712 have been used to prevent atheroma formation in a mouse model of atherosclerosis 31. Moreover, intelligent gelatinases stimuli nanoparticles have been used to co-deliver miR-200c and docetaxel to gastric cancer xenografts in mice, to simultaneously inhibit the growth of cancer stem cells and their more differentiated counterparts 32. In vitro experiments have demonstrated that hyaluronic acid-decorated polymeric nanoparticles can co-deliver doxorubicin and miR-542-3p to breast cancer cell lines 33 and mesoporous silica nanoparticles have been exploited to simultaneously deliver a miR-122 antagomir and small molecule inhibitors to hepatocellular carcinoma cells 34. Despite these successes, there are only limited reports of targeted miRNA delivery. In a study similar to our own, a polyarginine-PNA conjugate designed to inhibit miR-221 exhibited efficient cellular uptake and reduced miRNA expression in a breast cancer cells 35. However, this study was confined to in vitro observations and the polyarginine sequence is used as a cell-penetrating peptide rather than a tissue-specific targeting element. In a more technically advanced study, targeted nanoparticles decorated with a cyclic RGD peptide were used to deliver exogenous miR-296 to tumour vasculature in vivo, resulting in a significant decrease in microvessel formulation 36. Here, we provide the first report of targeted delivery of a miRNA inhibitor to the placenta.

Over recent years, a plethora of miRNAs have been proposed as putative regulators of trophoblast growth and function, and manipulation of miRNA expression using mimetics or antagomirs has confirmed their functional effects. Overexpression of mir-378a or miR-376c significantly enhanced proliferation, migration and invasion of the extravillous trophoblast (EVT)-like cell line HTR8/SVneo, and both mimetics promoted outgrowth of primary EVT from placental explants 37,38. Conversely, overexpression of miR-20a significantly inhibited proliferation, migration and invasion of the JEG-3 cell line, and inhibited the growth of JEG-3 tumour xenografts in nude mice 39. Inhibition of miR-21 reportedly reduced proliferation, migration and invasion of both JEG-3 and HTR-8 cells 40, and transfection of primary human term cytotrophoblasts with mimetics or antagomirs of miR-210 resulted in significant changes in mitochondrial respiration and oxygen consumption 41. Our ultimate aim is to manipulate expression of placental-specific miRNAs, to further reduce the risk of antagomirs causing off-target effects in other tissues, and to correct dysregulated miRNA expression that may underlie the pathology of pregnancy complications. A number of the same C19MC miRNAs are downregulated in gestational hypertension, pre-eclampsia and FGR, and disease-specific miRNAs have also been identified 20,21,42. As many of these miRNAs, including miR-145, have been detected in the peripheral blood of women with pregnancy complications 42, the potential for their use as biomarkers of disease and signposts for development of miRNA-specific personalised therapies also remains to be explored.

The downstream targets of miR-675 have already been characterised in the mouse placenta and include the *igfr1* gene 25. We have previously demonstrated a reduction in miR-675 expression in the human placenta in the third trimester 16, but its gene targets and function remain to be established. Of interest is our observation that placental miR-675 expression was quite varied in mice treated with the scrambled inhibitor conjugate (Figure 4H); placentas were randomly selected for analysis and expression level did not correlate with placental uterine horn position, the pregnant dam from which the placentas came, or individual fetal or placental weights. The significance of this level of biological variability is currently unknown.

We have also identified a number of downstream targets of miR-145 in the human placenta, including IGF receptor-1, cyclin D1 and p38 MAPK 16, all of which promote growth signalling. In other tissues miR-145 has been identified as a putative tumour suppressor gene 43 and as a negative regulator of angiopoietin-2 44, MUC-1 45 and ADAM-17 46. As these molecules have been shown to modulate implantation 47, trophoblast invasion 48 and spiral artery remodelling 49 respectively, miR-145 inhibition in the early stages of gestation has the potential to regulate multiple aspects of human pregnancy and placental development. Moreover, as miR-145 limits tumour angiogenesis 50,51, miR-145 inhibition may promote the normal physiological processes of decidual angiogenesis and uterine spiral artery remodelling which are key to pregnancy success in both humans and mice 52,53. These wide-ranging effects may highlight some of the ways in which miR-145 inhibition may have enhanced fetal growth in our study, without significantly increasing placental weight. Indeed, analysis of the relative area of the labyrinth and junctional zone is indicative of a possible growth-promoting effect of the miR-145 inhibitor conjugate within the junctional zone, although this requires further investigation.

It is evident from our in vivo data that there was inherent variability in miRNA inhibition following administration of the conjugates and there may be a reason to account for this. Unlike the human uterus, the mouse uterus is bicornuate and perfused in series by a bidirectional flow of maternal blood. Thus, the fetuses positioned at the top and bottom of the horn are likely to receive the highest plasma concentration of conjugate, whereas those positioned in the middle of the horn receive blood that has already passed through one or more placentas and may be somewhat depleted of conjugate. In our study, placentas were randomly selected for analysis, thus our data likely reflects this variability in dosing. In addition, the conjugate may only penetrate the cell layers closest to the placental vasculature, so it is unlikely that miRNA inhibition was achieved within every single placental cell. Finally, placental tissue was harvested at E18.5, two days after the final injection. Thus, we may have achieved significant miRNA knockdown at earlier time points in gestation, but miRNA expression may have subsequently normalised by the time of analysis. Despite these challenges, we still achieved a significant inhibition of miR-675 with our current dosing regimen.

These reasons may also explain why a significant increase in proliferation was not observed in the harvested mouse placentas despite miR-675 inhibition. Firstly, as the rate of conjugate delivery may have been variable, the basal rate of proliferation may have also varied between individual placentas or different regions of the same placenta, and as previously described, the maximal inhibitory effect may have been observed earlier in gestation. Indeed, if this were the case, it would correlate with our data from human placental explants, where the miRNA inhibitors had maximal effect in first trimester tissue but had no effect in term explants. Secondly, enhanced proliferation would lead to an increase in the total number of placental cells, as well as the number of Ki67 positive cells; thus, the proportion of immunostained nuclei compared to unstained nuclei may have remained the same. Finally, it would not be surprising that some additional regulatory mechanisms exist to prevent excessive placental (and therefore fetal) overgrowth.

We also observed that the scrambled inhibitor conjugate induced a non-significant increase in fetal weight, highlighted by the shift in fetal weight distribution; the authors acknowledge that this pilot study may not have been sufficiently powered to detect subtle increases in fetal weight and this outcome should be investigated in more detail. In previous studies, the off-target effects of siRNAs have been attributed to a number of causes, including regulation of unintended transcripts through partial sequence complementarity, induction of inflammatory responses via Toll-like receptor activation and perturbation of endogenous miRNA processing and function via saturation of the endogenous RNAi machinery 54. Saturation of exportin 5 activity, a component that is shared between the shRNA and microRNA processing pathways and is required for nuclear export of both molecules, has also been identified as potential cause of off-target effects 55. Our scrambled inhibitor conjugates may have acted through any of these mechanisms and although the outcome was enhanced fetal growth, this phenomenon requires further investigation. However, the off-target effects of miRNAs can be species-specific, highlighted by a comparison of the off-target transcripts regulated by three apolipoprotein B-specific siRNAs in mouse liver in vivo, and in cultured mouse and human liver tumour cells. Numerous common off-target transcripts were regulated by the siRNAs in mouse liver cells in vitro and *in vivo,* but there was only random overlap in the off-target transcripts regulated by the siRNAs when the mouse and human cell lines were compared 56. This was attributed to poor conservation of 3′ UTR sequences, and suggests that the off-target effects observed in our mouse study may not be the same as those occurring in humans.

In summary, we have demonstrated that homing peptide-miRNA inhibitor conjugates can be utilised to enhance placental growth; hence, it should now be possible to manipulate the expression of placental miRNAs that contribute to the development of pre-eclampsia and FGR. This could be achieved by two approaches: firstly, as demonstrated in our study, miRNAs that are highly expressed in the first trimester (such as miR-145 and miR-675), and are negative regulators of growth and development, could be targeted for inhibition in women identified as being at high risk of impaired placentation. Secondly, and likely more feasible, is that miRNAs which are highly expressed in the second and third trimester, and are negative regulators of different aspects of placental function, could be targeted for inhibition in women presenting with pre-eclampsia or fetal growth restriction, to treat the underlying pathophysiology. As more research is undertaken to understand the wide-ranging functions of miRNAs in the placenta, additional therapeutic targets will emerge, some of which may be placental-specific miRNAs. We therefore believe that the design of our novel therapeutics can easily be adapted to facilitate targeted manipulation of gene expression underlying compromised placental development and function.

## Supplementary Material

Supplementary figure S1.

## Abbreviations

3'UTRs: 3'-untranslated region
CTB: cytotrophoblasts
DMEM: Dulbecco's modified Eagle medium
EVT: extravillous trophoblast
FAM: 5(6)-carboxyfluorescein
FGR: fetal growth restriction
IGF-I: insulin-like growth factor-I
IGF-II: insulin-like growth factor-II
miR-145: microRNA-145
miR-675: microRNA-675
miRNA: microRNA.
