# Supplementary material for: Placental Homing Peptide-microRNA Inhibitor Conjugates for Targeted Enhancement of Intrinsic Placental Growth Signaling
Source: Theranostics. 2017 Jul 14;7(11):2940–55. doi: 10.7150/thno.18845 (PMC5562227; doi:10.7150/thno.18845)
Supplement: Supplementary file 1 — Supplementary figure S1. [file thnov07p2940s1.pdf]

**Supplementary Figure S1: Administration of a scrambled miRNA inhibitor does not alter pregnancy outcome.** Pregnant mice were intravenously injected with a fluorescently labelled, scrambled miRNA inhibitor (10 mg/kg), receiving either a single injection on E12.5, with tissue collection 24h later at E13.5 (short treatment), or three separate injections on E12.5, E14.5 and E16.5, with tissue collection at E18.5 (extended treatment). Control mice were injected with PBS (100µl) at the same time point(s). The following variables were measured: **[A, B]** fetal weight; **[C, D]** placental weight; **[E, F]** fetal/placental weight ratio; **[G, H]** number of fetuses per litter; **[I, J]**, number of resorptions per litter. Data points represent mean value per litter; horizontal line represents median; n= 3-5 mice per treatment group.

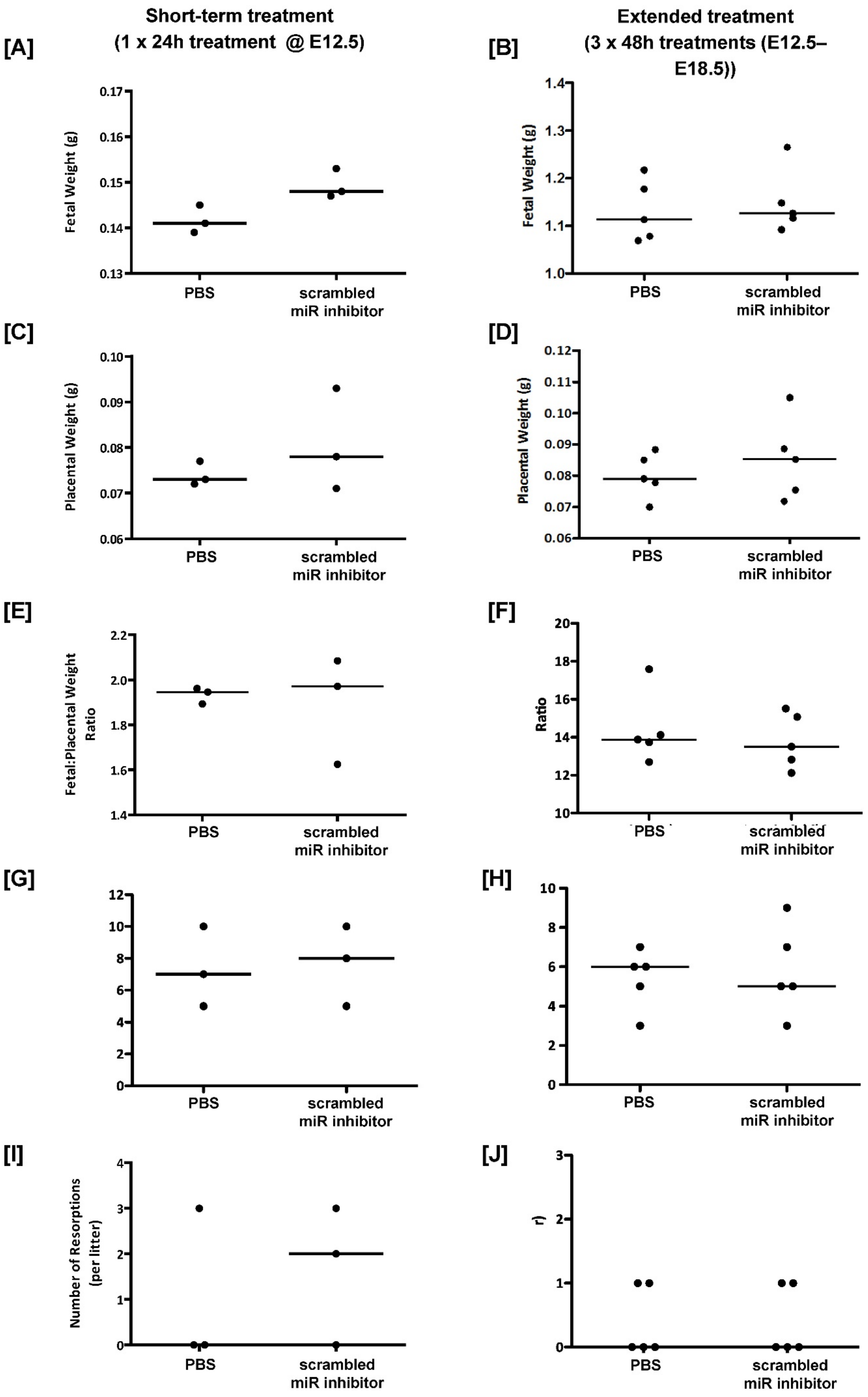

Short-term treatment
(1 x 24h treatment @ E12.5)
Extended treatment
(3 x 48h treatments (E12.5–E18.5))
[A]
[B]
[C]
[D]
[E]
[F]
[G]
[H]
[I]
[J]
Fetal Weight (g)
Placental Weight (g)
Fetal:Placental Weight Ratio
Ratio
Number of Resorptions (per litter)
r)
PBS
scrambled miR inhibitor
